# Microsatellite instability is biased in Amsterdam II-defined Lynch-related cancer cases with family history but is rare in other cancers: a summary of 1000 analyses

**DOI:** 10.1186/s12885-022-09172-5

**Published:** 2022-01-17

**Authors:** Hiroyuki Matsubayashi, Satomi Higashigawa, Yoshimi Kiyozumi, Takuma Oishi, Keiko Sasaki, Hirotoshi Ishiwatari, Kenichiro Imai, Kinichi Hotta, Yohei Yabuuchi, Kazuma Ishikawa, Tatsunori Satoh, Hiroyuki Ono, Akiko Todaka, Takeshi Kawakami, Hiromichi Shirasu, Hirofumi Yasui, Teichi Sugiura, Katsuhiko Uesaka, Hiroyasu Kagawa, Akio Shiomi, Nobuhiro Kado, Yasuyuki Hirashima, Yoshio Kiyohara, Etsuro Bando, Masashi Niwakawa, Seiichiro Nishimura, Takeshi Aramaki, Nobuaki Mamesaya, Hirotsugu Kenmotsu, Yasue Horiuchi, Masakuni Serizawa

**Affiliations:** 1 Division of Genetic Medicine Promotion, Shizuoka, Japan; 2 Division of Endoscopy and Genetic Medicine Promotion, Shizuoka Cancer Center, 1007, Shimonagakubo, Nagaizumi, Suntogun, Shizuoka, 411-8777 Japan; 3 Division of Pathology, Shizuoka, Japan; 4 Division of Gastrointestinal Oncology, Shizuoka, Japan; 5 Division of Hepato-Biliary-Pancreatic Surgery, Shizuoka, Japan; 6 Division of Colon and Rectal Surgery, Shizuoka, Japan; 7 Division of Gynecology, Shizuoka, Japan; 8 Division of Dermatology, Shizuoka, Japan; 9 Division of Gastric Surgery, Shizuoka, Japan; 10 Division of Urology, Shizuoka, Japan; 11 Division of Breast Surgery, Shizuoka, Japan; 12 Division of Interventional Radiology, Shizuoka, Japan; 13 Division of Thoracic Oncology, Shizuoka, Japan; 14 Department of Psychiatry and Behavioral Sciences, Tokyo Metropolitan Institute of Medical Science, Tokyo, Japan; 15 Division of Clinical Research Center, Shizuoka Cancer Center, Shizuoka, Japan

**Keywords:** Microsatellite, Cancer, Lynch syndrome, Genetic medicine, Companion diagnostics

## Abstract

**Background:**

Microsatellite instability (MSI) is a key marker for predicting the response of immune checkpoint inhibitors (ICIs) and for screening Lynch syndrome (LS).

**Aim:**

This study aimed to see the characteristics of cancers with high level of MSI (MSI-H) in genetic medicine and precision medicine.

**Methods:**

This study analyzed the incidence of MSI-H in 1000 cancers and compared according to several clinical and demographic factors.

**Results:**

The incidence of MSI-H was highest in endometrial cancers (26.7%, 20/75), followed by small intestine (20%, 3/15) and colorectal cancers (CRCs)(13.7%, 64/466); the sum of these three cancers (15.6%) was significantly higher than that of other types (2.5%)(*P* < 0.0001). MSI-H was associated with LS-related cancers (*P* < 0.0001), younger age (*P* = 0.009), and family history, but not with smoking, drinking, or serum hepatitis virus markers. In CRC cases, MSI-H was significantly associated with a family history of LS-related cancer (*P* < 0.0001), Amsterdam II criteria [odds ratio (OR): 5.96], right side CRCs (OR: 4.89), and multiplicity (OR: 3.31). However, MSI-H was very rare in pancreatic (0.6%, 1/162) and biliary cancers (1.6%, 1/64) and was null in 25 familial pancreatic cancers. MSI-H was more recognized in cancers analyzed for genetic counseling (33.3%) than in those for ICI companion diagnostics (3.1%)(*P* < 0.0001). Even in CRCs, MSI-H was limited to 3.3% when analyzed for drug use.

**Conclusions:**

MSI-H was predominantly recognized in LS-related cancer cases with specific family histories and younger age. MSI-H was limited to a small proportion in precision medicine especially for non-LS-related cancer cases.

## Background

Microsatellites are defined as 10 to 60 base pair regions which contain repeated multiple tandems consisting of 1 to 5 base pair motifs (≤ 10 bp) [1, 2] that are distributed widely throughout the genome. DNA repeats in the microsatellite loci are normally verified and maintained during cell division by the mismatch repair (MMR) function [3]. Impairment of a microsatellite system can render cells unable to regulate the length of microsatellites during cell division, a condition termed microsatellite instability (MSI). After multiple cycles of cell division, cells with an impaired MMR system will develop varying lengths in their microsatellite sequences.

MSI is a key marker to predict the effects of immune checkpoint inhibitors (ICIs) against several human cancers, as cancers with a high level of MSI (MSI-H) present increasing numbers of neoantigens that can be ICI targets [4, 5]. MSI is also a hypermutator phenotype that occurs in tumors with a deficient DNA mismatch repair function (dMMR) and therefore is a crucial screening factor for Lynch syndrome (LS), which has been diagnosed in 13–16% [6–8] of MSI-H cancers. As LS patients are at risk of developing multiple cancers, regular surveillance for their high-risk organs is performed to detect LS-related cancers in their early stages [9]. Hence, the detection of MSI in cancer has dual benefits in the genetic and oncological senses.

In this decade, MSI assays have become fully recognized among oncologists as a companion diagnostic or as a part of a multigene panel test [10–12] for judging indications of ICIs (PD-1 antagonist and PD-L1 antagonist). In December 2018, the Japanese national health insurance began covering MSI tests for cases with solid cancers refractory to the standard pharmacotherapy [13]. MSI can be analyzed using formalin-fixed paraffin-embedded cancer tissues [14] alone or in combination with non-neoplastic control DNA. The positivity of MSI or dMMR tends to be higher in LS-associated cancers, such as cancers in the colorectum, endometrium, small intestine, ureter, and renal pelvis [as defined in the Amsterdam II (AII) criteria] [8]. According to the revised Bethesda (rB) guidelines [7], which were established for indicating MSI testing, cancers of the stomach, pancreas, biliary tract, ovary, and brain and cutaneous neoplasms (keratoacanthoma and sebaceous gland adenoma) are also considered LS-associated cancers. In colorectal cancer (CRC) cases, MSI is also caused by the promoter methylation of *MLH1* (often coupled with *BRAF* V600E mutation) [15, 16], characterized by the CpG island methylator phenotype [17] independent from LS.

This study analyzed the MSI status in 1000 Japanese human cancers with a comparison between genetic medicine and precision medicine (companion diagnostics). It also analyzed the characteristics of the MSI-H cancers with special reference to the patients’ personal and familial cancer histories.

## Methods

### Patients

A consecutive 1000 cancer patients [519 males and 481 females, 62.6 ± 12.0 years old (y.o.)], managed in the Shizuoka Cancer Center from January 2013 to September 2020, were entered in this study (Table 1). Of the 1000 cancers, 778 were analyzed for MSI to examine the indication of ICI via companion diagnostics and 222 were analyzed to detect LS in genetic counseling. These cancers included CRCs (466 cancers), pancreatic cancers (162), endometrial cancers (ECs)(75), biliary tract cancers (64), gastric cancers (36), uterine cervical cancers (34), laryngeal-pharyngeal and esophagus cancers (30), skin cancers (27), ovarian cancers (24), neuroendocrine carcinomas (NEC) (22), small intestine cancers (15), thymic cancers (10), breast cancers (7), hepatic cancers (6), brain tumors (2), and others (20) (Table 1). Age and incidence of smoking was not significantly different between the LS-related and non-LS-related cancers, however incidence of smoking was significantly higher in the non-LS-related cancer group (57.1%, 89/156) than LS-related cancer group (47.0%, 397/844) (*P* = 0.02) (Table 1).

**Table 1:** Demographics of the patients with cancers analyzed for microsatellite instability

Cancer type	n	Male (%)		Age		Purpose of MSI test		Smoking		Drinking	
		n	%	mean SD	range	genetic counseling	companion diagnostics	n	%	n	%
LS (AII)-related cancers											
Colorectum	466	283	61%	62.4 ± 12.3	25–87	127	339	302	65%	239	51%
Endometrium	75	0	0%	57.9 ± 10.5	35–81	31	44	20	27%	14	19%
Small intestine	15	9	60%	68.6 ± 11.1	50–82	6	9	9	60%	6	40%
Renal pelvic/ureter	0	0	ー	ー	ー	ー	ー	ー	ー	ー	ー
Subtotal	556	292	53%	62.0 ± 12.1^§1^	25–87	164	392	331	60%^#1^	259	47%^*1^
LS (rB-AII)-related cancers											
Pancreas	162	86	53%	65.9 ± 9.9	30–84	29	133	76	47%	70	43%
Biliary tract	64	40	63%	66.3 ± 9.7	38–84	1	63	33	52%	38	59%
Stomach	36	22	61%	64.6 ± 9.7	39–81	18	18	29	81%	24	67%
Ovary	24	0	0%	57.9 ± 13.4	27–80	3	21	5	21%	6	25%
Brain	2	1	50%	49.0 ± 4.2	46–52	2	0	1	50%	0	0%
Subtotal	288	149	52%	65.0 ± 10.4^§2^	27–84	53	235	144	50%^#2^	138	48%^*2^
Non-LS-related cancers											
Uterine cervix	34	0	0%	53.9 ± 13.1	33–75	0	34	14	41%	15	44%
Laryngopharyngus and esophagus	30	24	80%	66.7 ± 10.0	45–83	0	30	28	93%	22	73%
Skin	27	15	56%	65.1 ± 12.7	34–87	0	27	17	63%	10	37%
Neuroendocrine	22	16	73%	64.2 ± 9.2	47–79	0	22	16	73%	16	73%
Thymus	10	6	60%	58.9 ± 12.8	35–72	0	10	5	50%	6	60%
Breast	7	0	0%	51.4 ± 6.5	41–60	0	7	4	57%	2	29%
Liver	6	4	67%	68.3 ± 7.4	60–81	0	6	4	67%	3	50%
Others	20	13	65%	54.3 ± 18.0	13–83	5	15	13	65%	15	75%
Subtotal	156	78	50%	60.5 ± 13.4^§3^	13–87	5	151	101	65%^#3^	89	57%^*3^
Total	1000	519	52%	62.6 ± 12.0	13–87	222	778	576	58%	486	49%

*MSI* microsatellite instability, *SD* standard deviation, *LS* Lynch syndrome

§AII: Amsterdam II criteria, #rB: revised Bethesda guidelines. ^§^1 + ^§^2 vs. ^#^3: not significant, ^§^1 vs. ^§^2: not significant, #1 + #2 vs. #3: *P* = 0.053, *1 + *2 vs. *3: *P* = 0.02

At the initial hospital visit, patients and their families filled out questionnaires concerning disease history, family history, and lifestyles. The nurses reconfirmed the content of the questionnaires by conducting 20–30 min interviews with each patient. LS-associated cancers as defined in the AII criteria consisted of CRC, EC, renal pelvic and ureteral cancers, and small intestine cancer. Those defined in the rB guidelines included additional tumors: gastric, ovarian, pancreatic, and biliary tract cancers, brain tumors, and two cutaneous neoplasms (sebaceous adenoma and keratoacanthoma) [18]. None of the patients or their families had been diagnosed with LS before the MSI testing by the genetic counseling and companion diagnostics.

A pathological evaluation of the cancer, including the histological type and stage, was determined in the resected materials when the surgery was performed, but when using biopsy samples, the staging was judged on the clinical images, including computed tomography and endoscopy. Pathological data were corrected from the diagnostic reports by expert pathologists with their own expertise. In CRC cases, within the histological conditions described by the rB guidelines, only histological type (mucinous/signet-ring differentiation) was evaluated, as the patterns of lymphocytic reaction and cancer growth were sometimes difficult to determine in the biopsy specimens. If information of onset age or cancer type were unclear for the AII criteria and the rB guidelines, we treated them as not informative.

### MSI analysis

#### MSI analysis for oncogenic purposes

To view indications of ICI, Pembrolizumab (Keytruda®, Tokyo, MSD Japan), 778 cancers were analyzed for MSI by companion diagnostics using only tumor DNA extracted from the archives of pathological samples during December 2018 and September 2020. The pathological specimens during this period were fixed at a suitable time in 10% neutral buffered formalin for less than 48 h, then embedded in paraffin and preserved at room temperature following the Japanese guidelines on the handling and storage of tissue samples [19]. Only a small proportion of the samples were archived prior to this period. This assay was entrusted to the SRL laboratory company (Tokyo, Japan). Tumor DNA was extracted with macroscopic dissection from series of 5–10 slices of 10 μm thick sections. MSI analysis was performed by using the MSI Kit (FALCO biosystems, Kyoto, Japan), which is equipped with five mononucleotide microsatellite markers (Promega panel: BAT-25, BAT-26, MONO-27, NR-21, and NR-24), following the manufacturer’s protocol [13, 20]. MSI-H was defined when the tumor DNA demonstrated instability in two or more markers, whereas microsatellite stability (MSS) was defined when only one or a null marker showed instability.

#### MSI analysis for screening lynch syndrome

A total of 222 cancers were analyzed for MSI in the genetic medicine clinics to screen for LS suspected because of the patient’s cancer history and family history and the tumors’ histology and multiplicity, among other factors. Sixty-seven cancer samples (30.2%) were obtained before 2018, when the pathological sample handling guidelines were implemented. The MSI analysis was entrusted to the laboratory company FALCO biosystems and performed by following the abovementioned protocol, except for the additional use of non-neoplastic DNA as a control [20] and the partial use of Bethesda panel markers (BAT-25, BAT-26, D2S123, D5S346, and D17S250) [21] until September 2015 (11 of 222 cancers).

### Statistical analysis

Statistical analyses were performed using JAMAevidence® and JMP ver.11.2.0 statistical software (SAS Institute Japan Ltd., Tokyo, Japan). A Fisher’s exact test was used to assess the categorical variables, and a Mann-Whitney U test was used to analyze the continuous variables. Multivariate analysis was performed by a logistic regression test. A value of *P* < 0.05 was considered statistically significant.

## Results

### MSI in various human cancers

As shown in Table 2, MSI-H was recognized in 98 of 1000 cancers (9.8%): 26.7% (20/75) of ECs, 20% (3/15) of small intestine cancers, 16.7% (1/6) of hepatic cancers, 13.7% (64/466) of CRCs, and 10% (1/10) of thymomas. In LS-associated cancers defined by the rB guidelines, MSI-H was very rare in pancreatic (0.6%, 1 of 162) and biliary tract cancers (1.6%, 1/64). The 25 cases of familial pancreatic cancer were completely MSS. In other cancer categories, one prostate cancer and one adrenocortical cancer were MSI-H. From the 1000 cancer MSI tests, only one case of CRC was returned with an inconclusive result; however, it was compensated with a preserved MMR protein analyzed by immunohistochemistry. Incidence of MSI-H was significantly higher in genetic counseling group than in companion diagnostics group in CRC (41.7% vs. 3.2%, *P* < 0.0001), endometrial cancer (45.2% vs.13.6%, *P* = 0.003), in ovarian cancer (66.7% vs. 0%, *P* = 0.011), in the subtotal of LS (AII)-related cancers (42.1% vs. 4.6%, *P* < 0.0001), and in the subtotal of non-LS-related cancers (40.0% vs. 2.6%, *P* = 0.011) (Table 2).

**Table 2:** Personal and family cancer history and microsatellite instability of the patients with LS-related and non-LS-related cancer

Cancer type	n	Meeting rB GLs		Meeting AII criteria	No. of any cancer			No. of LS (rB)-related cancer			Family history of the same cancer				MSI-H				
	total	patient	family		in patient past history	in FDR	in SDR and TDR	in patient past history	in FDR	in SDR and TDR	in FDR	%	in SDR and TDR	%	n	%	% in GC (n)	% in CD (n)	*P* value (GC vs.CD)
LS (AII)-related cancers																			
Colorectum	466	259	116	40	0.3 ± 0.8	1.1 ± 1.1	0.7 ± 1.2	0.2 ± 0.7	0.7 ± 0.9	0.4 ± 0.9	132	28.3%	50	10.7%	64	13.7%	41.7% (53/127)	3.2% (11/339)	< 0.0001
Endometrium	75	7	16	7	0.2 ± 0.6	1.2 ± 1.1	1.0 ± 1.6	0.1 ± 0.4	0.6 ± 0.8	0.6 ± 1.0	5	6.7%	4	0%	20	26.7%	45.2% (14/31)	13.6% (6/44)	0.003
Small intestine	15	2	1	0	0.6 ± 0.7	1.0 ± 0.9	0.5 ± 0.7	0.3 ± 0.6	0.3 ± 0.6	0.2 ± 0.4	0	0%	0	5.3%	3	20.0%	33.3% (2/6)	11.1% (1/9)	0.525
Renal pelvic/ureter	0	ー	ー	ー	ー	ー	ー	ー	ー	ー	ー	ー	ー	ー	ー	ー	ー	ー	ー
Subtotal	556	268	133	47	0.3 ± 0.8	1.1 ± 1.1^¶1^	0.7 ± 1.2	0.2 ± 0.7	0.7 ± 0.9^§1^	0.4 ± 0.9	137	24.6%	54	9.7%	87	15.6%^*1^	42.1% (69/164)	4.6% (18/392)	< 0.0001
LS (rB-AII)-related cancers																			
Pancreas	162	9	22	0	0.3 ± 0.6	1.1 ± 1.1	0.8 ± 1.3	0.2 ± 0.5	0.7 ± 0.9	0.5 ± 1.1	25	15.4%	11	6.8%	1	0.6%	0% (0/29)	0.8% (1/133)	1.000
Biliary tract	64	2	11	0	0.2 ± 0.5	1.1 ± 1.2	0.4 ± 0.9	0.1 ± 0.3	0.5 ± 0.9	0.2 ± 0.5	2	3.1%	0	0%	1	1.6%	0% (0/1)	1.6% (1/63)	1.000
Stomach	36	4	11	2	0.5 ± 0.7	2.2 ± 1.6	0.8 ± 1.1	0.4 ± 0.7	1.7 ± 1.4	0.5 ± 0.9	16	44.4%	8	22.2%	1	2.8%	5.6% (1/18)	5.6% (1/18)	1.000
Ovary	24	1	3	0	0.4 ± 0.8	0.9 ± 1.4	0.9 ± 1.4	0.2 ± 0.6	0.7 ± 1.4	0.4 ± 0.9	0	0%	0	0.0%	2	8.3%	66.7% (2/3)	0% (0/21)	0.011
Brain	2	0	0	0	0.5 ± 0.7	0	0.5 ± 0.7	0	0	0.5 ± 0.7	0	0%	1	50.0%	0	0%	0% (0/2)	ー	ー
Subtotal	288	16	47	2	0.3 ± 0.6	1.2 ± 1.3^¶2^	0.7 ± 1.2	0.2 ± 0.5	0.8 ± 1.1^§2^	0.4 ± 0.9	43	14.9%	20	6.9%	5	1.7%^*2^	5.7% (3/53)	1.3%(3/235)	0.078
Non-LS-related cancers																			
Uterine cervix	34	1	3	0	0.2 ± 0.5	0.6 ± 0.7	1.4 ± 1.4	0.1 ± 0.2	0.4 ± 0.6	0.8 ± 1.1	0	0%	0	0%	0	0%	ー	0% (0/34)	ー
Laryngopharyngus and esophagus	30	0	0	0	0.4 ± 1.0	0.9 ± 1.2	0.3 ± 0.7	0.1 ± 0.3	0.5 ± 0.7	0.1 ± 0.3	3	10.0%	1	3.3%	2	6.7%	ー	6.7% (2/30)	ー
Skin	27	1	1	0	0.6 ± 0.9	0.9 ± 0.8	0.7 ± 1.1	0.3 ± 0.5	0.6 ± 0.8	0.3 ± 0.7	1	3.7%	0	0%	0	0%	ー	0% (0/27)	ー
Neuroendocrine	22	1	0	0	0.1 ± 0.4	0.9 ± 1.1	0.3 ± 0.6	0.1 ± 0.3	0.3 ± 0.5	0.1 ± 0.4	0	0%	0	0%	0	0%	ー	0% (0/22)	ー
Thymomus	10	0	0	0	0.3 ± 0.5	1.2 ± 0.9	1.1 ± 1.3	0	0.9 ± 0.9	0.5 ± 0.7	0	0%	0	0%	1	10.0%	ー	10.0% (1/10)	ー
Breast	7	0	0	0	0	0.9 ± 0.9	1.3 ± 1.8	0	0.3 ± 0.5	1.0 ± 1.2	1	14.3%	0	0%	0	0%	ー	0% (0/7)	ー
Liver	6	0	0	0	0.5 ± 0.8	0.3 ± 0.5	0.2 ± 0.4	0.2 ± 0.4	0	0.2 ± 0.4	0	0%	0	0%	1	16.7%	ー	16.7% (1/6)	ー
Others	20	1	3	1	0.4 ± 0.7	1.1 ± 1.1	1.1 ± 1.4	0.3 ± 0.6	0.6 ± 0.8	0.7 ± 1.3	0	0%	1	5.0%	2	10.0%	40.0%(2/5)	0% (0/15)	0.053
Subtotal	156	4	7	1	0.3 ± 0.7	0.8 ± 1.0^¶3^	0.8 ± 1.2	0.1 ± 0.4	0.5 ± 0.7^§3^	0.4 ± 0.9	5	3.2%	2	1.3%	6	3.8%^*3^	40.0% (2/5)	2.6% (4/151)	0.011
Total	1000	288	187	50	0.3 ± 0.7	1.1 ± 1.1	0.7 ± 1.2	0.2 ± 0.6	0.7 ± 0.9	0.4 ± 0.9	185	18.5%	76	7.6%	98	9.8%	33.3% (74/222)	3.1% (24/778)	< 0.0001

*LS* Lynch syndrome, *SD* standard deviation, *rB GLs* revised Bethesda guidelines, *AII* Amsterdam II, *FDR* first degree relative, *SDR* second degree relative, *TDR* third degree relative, *MSI-H* high frequency of microsatellite instability, *GC* genetic counseling, *CD* companion diagnostics

#1 + #2 vs. #3: not significant, #1 vs. #2: not significant, ¶1 + ¶2 vs. ¶3: *P* = 0.003, ¶1 vs. ¶2: not significant, §1 + §2 vs. §3: *P* = 0.004. §1 vs. §2: not significant, *1 vs. *2: *P* < 0.0001, *1 vs. *3: *P* < 0.0001

### MSI and cancer histories

The incidence of MSI-H was highest in the AII criteria-defined LS-related cancer group (15.6%, 87/556), which was significantly higher than both other LS-related cancers defined by the rB guidelines (1.7%, 5/288)(OR: 10.50, 95% CI: 4.33–25.43, *P* < 0.0001) and non-LS-related cancer groups (OR: 4.64, 95% CI: 2.03–10.58, *P* < 0.0001) (Table 2**)**. In these three groups, the number of the patients’ past cancers (excluding the cancer analyzed for MSI) was similar, either for any cancer type or for LS (rB)-related cancer types. However, the number of cancers developed in the FDRs was significantly higher in LS (rB)-related cancer patients (mean ± standard deviation: 1.1 ± 1.2) than in non-LS-related cancer patients (0.8 ± 1.0) (*P* = 0.003). This trend was commonly seen in the number of LS (rB)-related cancers in the FDRs (0.7 ± 1.0 in LS-related cancer patients vs. 0.5 ± 0.7 in non-LS-related cancer patients) (*P* = 0.004, Table 2) despite the similar ages of the patients among the three groups (Table 1).

### Factors associated with MSI-H analyzed in 1000 cancers

Factors associated with MSI-H were determined in all 1000 cases using univariate and multivariate analyses (Table 3). MSI-H was significantly more recognized in younger patients (≤50 y.o.)[OR: 1.88, 95% confidence interval (95% CI): 1.17–3.01, *P* = 0.01]; cancer types were defined by the AII criteria (OR: 7.30, 95% CI: 3.88–13.72, *P* < 0.0001) and the rB guidelines (OR: 3.06, 95% CI: 1.24–6.95, *P* = 0.005). The incidence of MSI-H was nearly ten times as often in cancers in LS-suspected patients (33.3%, 74 of 222) than in those in which MSI was analyzed for oncological purposes (3.1%, 24 of 778) (*P* < 0.0001). Within the factors considered statistically significant by univariate analysis, LS-related cancer types categorized by AII criteria (OR: 9.56, 95% CI: 4.09–28.05, *P* < 0.0001) and cases who underwent MSI for the diagnosis of LS (OR: 15.01, 95% CI: 8.91–26.17, *P* < 0.0001) were signified by multivariate analysis (Table 3).

**Table 3:** Clinical and demographic factors analyzed for the association with MSI-H cancer (*n* = 1000)

	n	MSI positive		Univariate analysis		Multivariate analysis	
		n	%	Odd’s ratio (95% confidence interval)	*P* value	Odd’s ratio (95% confidence interval)	*P* value
Age							
≤ 50y.o.	179	27	15.1%	1.88 (1.17–3.01)	0.01		
> 50y.o	821	71	8.6%				
Gender							
Male	519	42	8.1%	0.67 (0.44–1.02)	0.07		
Female	481	56	11.6%				
HBsAg and HCVAb							
Both (−)	950	92	9.7%	0.72 (0.26–2.02)	0.54		
Either (+)	31	4	12.9%				
Smoker							
Yes	576	58	10.1%	1.10 (0.71–1.64)	0.83		
No	424	40	9.4%				
Drinker							
Yes	486	43	8.8%	0.81 (0.53–1.23)	0.34		
No	514	55	10.7%				
Cancer type							
LS(AII)-related cancers§	556	87	15.6%	7.30 (3.88–13.72)	< 0.0001	9.56 (4.09–28.05)	< 0.0001
Others	444	11	2.5%				
LS(rB)-related cancers#	844	92	10.9%	3.06 (1.34–6.95)	0.005		
Others	156	6	3.8%				
Purpose of MSI test							
Genetic counseling	222	74	33.3%	15.71 (9.63–25.62)	< 0.0001	15.01 (8.91–26.17)	< 0.0001
Companion diagnostics	778	24	3.1%				

*MSI-H* high frequency of microsatellite instability, *AII* Amsterdam II criteria, *rB* revised Bethesda guidelines.LS(AII)-related cancers consist of colorectal cancer, endometrial cancer, renal pelvic and ureteral cancers, and small intestine cancer, and LS(rB)-related cancers include additional tumors; gastric cancer, ovarian cancer, pancreatic cancer, biliary tract cancer, brain tumor, and two cutaneous neoplasms (sebaceous adenoma and keratoacanthoma)

### Factors associated with MSI-H in colorectal cancers

In total, MSI-H was recognized in 13.7% (64/466) of CRCs and was significantly more recognized in the CRCs of younger patients (≤50 y.o.) (OR: 2.11, 95% CI: 1.17–3.82, *P* = 0.02) meeting the AII criteria (OR: 5.96, 3.00–11.87, *P* < 0.0001) and rB guidelines (OR: 3.66, 95%CI: 1.93–6.94, *P* < 0.0001) than in the other patients (Table 4). Similarly, CRCs in patients with increasing numbers of relatives with a history of cancer showed high risks of MSI-H. The ORs of family histories of rB guideline-defined LS cancers showed higher values compared with those of family histories of any cancer (Table 4). CRCs located in the right side of the colon (OR: 4.88, 95% CI: 2.80–8.51, *P* < 0.0001), those diagnosed at an early stage (OR: 3.21, 95% CI: 1.55–6.65, *P* = 0.003), and synchronous and/or metachronous multiple CRCs (OR: 3.31, 95% CI: 1.70–6.47, *P* = 0.001) were also statistically significant factors of MSI-H. The incidence of MSI-H analyzed in genetic medicine (41.7%, 53/127) was more than ten times as often as that analyzed for oncological purposes (3.2%, 11 of 339) (*P* < 0.0001). Within the statistically significant factors analyzed by the univariate test, CRC cases in first-degree relatives (FDRs)(OR: 9.98, 95% CI: 1.47–205.1, *P* = 0.046), CRCs located in the right side of the colon (OR: 5.21, 95% CI: 2.61–10.81, *P* < 0.0001), and cases who underwent genetic counseling (OR: 24.98, 95% CI: 10.07–70.08, *P* < 0.0001) were determined as independent significant factors by multivariate analysis (Table 4).

**Table 4:** Factors associated with MSI-H in colorectal cancers (*n* = 466)

	n	MSI positive		Univariate analysis		Multivariate analysis	
		n	%	Odd’s ratio (95% confidence interval)	*P* value	Odd’s ratio (95% confidence interval)	*P* value
Patient							
Age							
≤ 50y.o.	86	19	22.1%	2.11 (1.17–3.82)	0.02		
> 50y.o	380	45	11.8%				
Gender							
Male	283	36	12.7%	0.81 (0.48–1.37)	0.49		
Female	183	28	15.3%				
Personal and/or family history							
AII criteria							
(+)	40	17	42.5%	5.96 (3.00–11.87)	< 0.0001		
(−)	426	47	11.0%				
rB guidelines							
(+)	259	51	19.7%	3.66 (1.93–6.94)	< 0.0001		
(−)	207	13	6.3%				
≥ 3 LS(rB)-related cancers# in FDR, SDR and TDR							
(+)	51	17	33.3%	3.91 (2.03–7.55)	0.0001		
(−)	415	47	11.3%				
≥ 3 any cancers in FDR, SDR and TDR							
(+)	120	27	22.5%	2.43 (1.41–4.18)	0.002		
(−)	346	37	10.7%				
Colorectal cancer patient in FDR							
(+)	132	30	22.7%	2.60 (1.52–4.44)	0.001	9.98 (1.47–205.1)	0.046
(−)	334	34	10.2%				
Colorectal cancer							
Location							
Right-side colon	155	42	27.1%	4.88 (2.80–8.51)	< 0.0001	5.21(2.61–10.81)	< 0.0001
Left-side colon	311	22	7.1%				
Stage							
Early	39	12	30.8%	3.21 (1.55–6.65)	0.003		
Advanced	427	52	12.2%				
Multiplicity (simultaneous + metachronous)							
(+)	49	15	30.6%	3.31 (1.70–6.47)	0.001		
(−)	417	49	11.8%				
Purpose of MSI test							
Genetic counseling	127	53	41.7%	21.36 (10.74–42.40)	< 0.0001	24.98 (10.07–70.08)	< 0.0001
Companion diagnostics	339	11	3.2%				

*MSI-H* high frequency of microsatellite instability, *AII* Amsterdam II, *rB* revised Bethesda, *LS* Lynch syndrome

### MSI-H in endometrial cancer

Because the incidence of MSI-H was highest in ECs (26.7%, 18/74) (Table 2), we analyzed factors associated with MSI-H similar to those for CRCs. Incidence of MSI-H was significantly higher in genetic counseling group than in companion diagnostics group (45.2% vs. 13.6%, *P* = 0.003). Incidence of MSI-H showed a higher trend in younger patients (30% in ≤50 y.o. vs. 25.5% in > 50 y.o.) and cases meeting the AII criteria (42.9% in cases fulfilling AII criteria vs. 25% in the other cases); however, none of these statistics, including other family history analyses done in the CRC cases, reached significant values. Additionally, neither histological type or FIGO clinical stage showed an association with MSI-H; incidence of MSI-H was 27.2% (15/55) in endometrioid carcinoma and 25.0% (5/20) in other histological types of carcinoma (*P* = 1.000), and 28.6% (10/35) in FIGO I-II and 25.0% (10/40) in FIGO III-IV (*P* = 0.797).

## Discussion

In the current study, we analyzed the MSI status of 1000 human cancers in a tertiary Japanese cancer center and examined the incidence of MSI-H in various cancers in association with several demographic and clinical factors. The results demonstrated a significantly higher incidence of MSI-H in the LS-related cancers categorized by the AII criteria but not by the rB guidelines, in younger patients (≤50 y.o.), and in patients who underwent genetic counseling rather than simple companion diagnostics. MSI status was not associated with other patient demographics, such as smoking, drinking, and hepatitis virus serum markers (Table 3). These data were informative as, so far, MSI data from a large number of cancer cases has not been fully reported in reference to family histories, particularly in Asian countries.

MSI status has recently been analyzed in a variety of cancers in Western countries by international genome projects [The Cancer Genome Atlas (TCGA) [22, 23]/International Cancer Genome Consortium (ICGC) [24]/Therapeutically Applicable Research to Generate Effective Treatments (TARGET) [22]] and as a part of precision medicine (e.g., Memorial Sloan Kettering-integrated mutation profiling of actionable cancer targets: MSK-IMPACT [8]). In these genome projects, the prevalence of MSI-H was highest in EC (28.3% [25]–31.4% [22]), followed by gastric (21.9% [25]), colon (19.7% [22]), rectal (5.7% [22]–9.2% [25]), adrenocortical (4.3% [22]–5.4% [25]), esophageal (3.3% [25]), and ovarian cancers (3.2% [25]). MSK-IMPACT [8], which focuses on the more advanced stages of cancers, demonstrated relatively lower incidence; however, the data combined MSI-low (MSI-L) with MSI-H: 29.8% in small intestine cancer, 22.7% in EC, 16.6% in CRC, 7.8% in esophageal cancer, and 5.0% in gastric cancer. These data trends resemble the current data in overall and in companion diagnostics, respectively, although the current data showed a trend with lower incidence (Table 2). Generally, MSI in LS-related cancers has been reported in lower frequencies in Asian countries; for example, 17.3–25.7% [26–28] in EC, 4.3–10.0% in CRC [26, 29–31], and 2.3–9.3% in gastric cancer [26, 29, 32], suggesting an ethnic deviation. This is also reflected in the lower incidences of LS in Asian countries (2.9% [33]–4.4% [28] in EC and 0.6% [34]–0.7% [35] in CRC).

Non-LS-related cancers, such as cervical and skin cancer and NEC, showed a significantly lower incidence of MSI-H (3.8%) compared with the LS (rB)-related cancer groups (10.9%)(*P* = 0.005) and the LS (AII)-related cancer group (15.6%)(*P* < 0.0001) (Table 2). This group showed a significantly lower incidence of personal and familial cancer histories than the LS-related cancer groups (Table 2), despite a significantly higher ratio of smoking (65%) and drinking (57%)(Table 1), suggesting that the majority of these cancers developed with chromosomal instability [36]. Furthermore, LS-related cancers categorized under the rB guidelines but not the AII criteria, such as gastric, pancreaticobiliary, and ovarian cancers and brain tumors showed significantly lower MSI-H incidence (1.7%) than the LS-related cancers defined in the AII criteria (15.6%)(*P* < 0.0001) (Table 2), which is also compatible with TCGA [22, 25]. This is despite the similar levels of patients’ personal histories and family histories of cancer between these two groups. For pancreaticobiliary cancers, the higher indication of ICI was expected because of their aggressive biological behavior and poor prognosis but much improved in cases with MSI-H and treated by ICI [37]. However, the current cases, including 25 familial pancreatic cancers and 2 familial biliary cancers, showed a very low MSI-H incidence (0.6–1.6%), similar to the low level of tumor mutation burden (0.5–2.2/Mb) demonstrated in our previous study [34]. In pancreatic and ovarian cancers, PARP inhibitors are effective when the patients harbor germline variants in the genes associated with homologous recombination pathways. In the K-*ras* wild-type pancreatic cancer cases, although in less than 10% of the cases, the mTOR inhibitors may have potential therapeutic importance since they often harbor RAS-MAPK pathway-activating alterations and elevated phosphorylation mTOR pathway proteins [38]. As the benefits from MSI companion diagnostics are limited in several cancer types, suitable molecular target agents should be selected by the specific genetic alterations detected in the cancer gene panel testing.

To date, the dMMR of CRCs has been analyzed in several studies. Although most of them were screened by MMR protein immunohistochemistry or by a partial combination with MSI, the concordance with MSI-H and loss of the MMR protein were reported to be very high in CRCs (> 99% [39] and κ = 0.81 [40]). When universal screening was conducted on CRCs, the incidence of dMMR varied in a small range by country [7.3% in Slovenia [41], 8.6% in Spain [42], 9.6–12.6% in the U.S. (similar in blacks, Hispanics and non-Hispanic whites) [40], 9.8% in France [43], 11.7% in Italy [44], 15.0% in Switzerland [45], 16.9% in Australia [46], and 4.3–10.0% in Asian countries [26, 29–31]]. dMMR is characteristically recognized in CRCs in younger patients [40, 44, 46], in multiple cancer cases [40], in the proximal colon [44], and with specific pathological features [47]; however, these findings were not always statistically significant, probably due to a low number of cases. The current 466 CRC cases, although not a universal series, showed a significantly higher incidence of MSI-H in cases meeting the AII criteria (OR: 5.91) and the rB guidelines (OR: 3.71), with increasing risk according to the number of family history of cancers, and in CRCs located in the right side of the colon (OR: 4.89), in early stage cancers (OR: 3.18), and in younger patients (≤50 y.o.)(OR: 1.97) (Table 4). With the reported knowledge of MSI-H CRCs in mind, we selected patients for genetic counseling, in whom we detected a significantly higher incidence of MSI-H (41.7%) compared with those who underwent a companion test (3.2%). We judge that this large difference is not dependent on the methodology of MSI testing between genetic medicine (using normal tissue DNA as a control) and companion diagnostics (tumor only) because tumor-only MSI analysis demonstrated a nearly perfect concordance with the standard method of measuring the quasi-monomorphic variation range of the PCR products of Promega microsatellite markers [48]. Younger age and family history in MSI-H cancer suggested the inherited predisposition of the CRC, or LS, existed in some proportion, hence the needs for the genetic counseling is emphasized.

In Japan, in December 2020, the national health insurance system was revised to support the analysis of MSI for surgically-resected advanced CRCs, in addition to unresected cases, to investigate the indication of ICI as adjuvant therapy. MSI testing was thus made available in ≥85% of surgically-resected and far-advanced CRCs [49]. Since 2018, the National Comprehensive Cancer Network (NCCN) guidelines recommend universal tumor screening using an MSI test or MMR immunohistochemistry in all newly diagnosed CRCs for detecting LS patients [50]. It is easily suggested that, with these nationwide policies, diagnoses of LS will increase in the near feature. With the current data in mind, other AII-defined cancers (EC and small intestine cancer) also need MSI testing. As of now, universal dMMR screening is not, at least not unanimously, thought to be cost-effective [51, 52]. Genetic medicine for cancer patients requires a sense of balance in mental, physical, temporal, and economic burdens on patients and needs to be conducted in more efficient ways to achieve systemic care of the patients and their families.

The current study had several limitations due to its retrospective design and being done in a single cancer center hospital. The study subjects were a mixture of patients who had undergone genetic medicine and companion diagnostics, and the ratio of such testing may differ by institution. Family histories were obtained from interviews with the patients and their families so that they may be lacking in detail, especially concerning onset age and exact cancer type, leading to the lower evaluation of cases meeting the AII criteria and the rB guidelines. The pathological evaluations of lymphocytic response around CRC [47] are excluded due to the biopsy specimens included in some proportion; therefore, fewer cases may have been evaluated as meeting the rB guidelines. Besides, a cancer of renal pelvis and ureter, one of LS-related cancers in the AII criteria, was not included in this study.

In conclusion, MSI-H was found to strongly deviate in LS-related cancers defined in the AII criteria with significant associations to personal and family cancer histories and younger age. LS screening needs to be performed in an efficient way in both genetic and precision medicine.

## Data Availability

The data analyzed during the current study are available from the corresponding author on reasonable request.

## Abbreviations

MMR: Mismatch repair
MSI: Microsatellite instability
ICI: Immune checkpoint inhibitor
MSI-H: High level of MSI
dMMR: Deficient DNA mismatch repair
LS: Lynch syndrome
CRC: Colorectal cancer
y.o.: Years old
EC: Endometrial cancer
NEC: Neuroendocrine carcinoma
AII: Amsterdam II
rB: Revised Bethesda
MSS: Microsatellite stability
OR: Odd’s ratio
95%CI: 95% confidence interval
FDR: First-degree relative
SDR: Second-degree relative
TDR: Third-degree relative
TCGA: The Cancer Genome Atlas
ICGC: International Cancer Genome Consortium
TERGET: Therapeutically Applicable Research to Generate Effective Treatments
MSK-IMPACT: Memorial Sloan Kettering-integrated mutation profiling of actionable cancer targets
NCCN: National Comprehensive Cancer Network
